# Gradual wiring of olfactory input to amygdala feedback circuits

**DOI:** 10.1038/s41598-020-62457-2

**Published:** 2020-04-03

**Authors:** Livio Oboti, Katie Sokolowski

**Affiliations:** 1 Center for Neuroscience Research, Children’s National Health System, Washington, DC 20010 USA; 2 Present Address: Department of Behavioral Physiology, Humboldt Universität zu Berlin, Berlin, 10115 Germany

**Keywords:** Synaptic development, Amygdala

## Abstract

The amygdala facilitates odor driven behavioral responses by enhancing the saliency of olfactory signals. Before this processing, olfactory input is refined through the feedback provided by amygdala corticofugal projection (ACPs). Although the saliency of odor signals is subject to developmental changes, the stage at which this cortical feedback first occurs is not known. Using optogenetically-assisted intracellular recordings of the mouse cortical amygdala, we identified changes in the electrophysiological properties of ACPs at different developmental stages. These were consistent with a decrease in neuronal excitability and an increase in the amount of incoming accessory olfactory bulb (AOB) inputs, as confirmed by estimates of release probability, quantal size and contact number at the AOB-to-ACP synapse. Moreover, the proportion of ACPs activated in response to odors was dependent on the stage of development as revealed by c-Fos expression analysis. These results update standard accounts of how the amygdala processes social signals by emphasizing the occurrence of critical periods in the development of its sensory gating functions.

## Introduction

The amygdala is considered to be primarily involved in the emotional connotation of sensory stimuli. This appears to be true for behaviorally salient sensory input after sensory processing circuits have established functional interconnections with the amygdala, which typically occurs during postnatal development^1–3^. The late display of malaise-induced odor aversion^1^ and conditioned odor avoidance in early postnatal rats^2^ are clear examples of emotional “tagging” which have been hypothesized to develop gradually, together with the maturation of olfactory-amygdala circuits^3^. Natural responses to social odors develop at later stages, as demonstrated in both mice and rats during the first postnatal weeks^4,5^. Nonetheless, direct physiological evidence supporting a delayed wiring of amygdala-olfactory circuits has yet to be provided.

The posteromedial cortical nucleus of the amygdala (PmCo) is the primary cortical area of the accessory olfactory system (AOS), a sensory pathway specialized in processing social odor information^6^. The PmCo receives direct input from AOB excitatory output neurons (i.e. mitral cells, or MCs^7^) and in turn gives rise to feedback projections to AOB inhibitory circuits^8^, which are responsible for shaping the activity patterns of AOB MCs.

Due to this reciprocal connectivity, the PmCo likely plays a relevant role in gating sensory input to value-encoding amygdala circuits. However, age-dependent changes in corticofugal circuits processing olfactory input have not been described.

In this study, we conducted an electrophysiological and morphological characterization of the amygdala corticofugal projections (ACPs) to the AOB, with a focus on their afferent inputs during postnatal maturation. We show that ACPs receive direct input from AOB MCs. By analyzing the evoked responses to MC afferents together with other physiological parameters of the MC-ACP synapse, we provide evidence of a gradual establishment of AOB-to-PmCo connectivity during the first 3–5 weeks of postnatal development. Furthermore, mature – but not juvenile – mice exposed to urine derived odors demonstrate an increased expression of the neuronal activity marker c-Fos in both the PmCo and ACP neurons. Overall, these results reveal a delayed innervation of amygdala feedback circuits by primary sensory afferents. These findings appear to represent a novel synaptic mechanism underlying the differential processing of social odors occurring at different postnatal stages.

## Results

### Developmental changes in ACP electrical properties

To gain initial insight into the developmental factors possibly affecting ACP electrophysiological properties, we conducted a morphological and electrophysiological analysis of ACPs at different postnatal stages (N = 104 cells in total).

**Figure 1:**
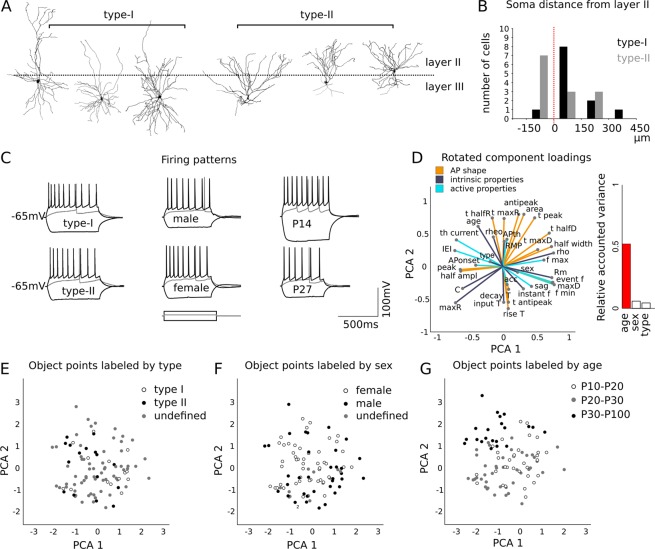
Analysis of ACP electrophysiological parameters. (**A**) A total of 104 ACPs were recorded in the PmCo. Recorded cells were analyzed using three grouping variables: putative cell subtype, sex and age. (**B**) Two putative ACP subtypes were identified based on the relative complexity of apical and basal dendritic arbors and on their location relative to the PmCo layer II-III boundary. (**C**) Traces showing hyperpolarization (bottom traces), threshold (gray intermediate traces) and depolarization (top traces) responses of ACP pairs representative of the three categories: cell subtype, sex and age (current clamp protocol: injected current range, from −200 to +190 pA, step 10 pA, duration 600 ms). No differences in firing patterns or other electrophysiological parameters were observed among cells, except for those of different age. (**D**) Categorical principal component analysis (CATPCA) was used to identify factors underlying the variability of recording parameters based on cell type (**E**), sex (**F**) and age (**G**). The plot in D shows the variable loadings into the reduced dimensions individuated by CATPCA, in the rotated variable space (see Table 1 for the complete list of variables used). In the histogram the contribution of each grouping variable to the total variance of the sample is reported. (**E–G**) Recordings labeled by subtype (**E**), sex (**F**) and age (**G**). Gray data points in (**E,F**) were not part of the categorization.

We began by surveying the most representative morphological cell types in our sample by analyzing neuronal 3D reconstructions obtained from biocytin filled neurons (see methods; N = 25 ACPs, Fig. 1A). At first glance, ACP morphology appeared heterogeneous in terms of apical dendritic arborization and soma localization relative to the layer II-III boundary. Deeply localized cells displayed large apical dendritic arbors while more superficially located neurons showed complex basal dendrites and limited apical skirts (Fig. 1A,B).

Based on these features, we categorized neurons into two distinct putative types (type I, deep; type II, superficial), which were reminiscent of piriform pyramidal cells^9^.

However, when we compared ACPs by type, sex or age, we found differences in electrical properties only relative to age (Fig. 1C). To conclusively rule out the presence of type-related differences and to evaluate the contribution of all electrophysiological parameters, we performed a factor analysis to extract principal components using both categorical and scalar variables (see methods, Table 1). Biplot analysis of a two-dimensional variable space confirmed a clear effect of *age* without any strong correlation with the other factors considered (cell type and sex), suggesting their contribution to the total variance is low (Fig. 1D).

**Table 1:** Variables used for the principal component analysis (Fig. 1).

Variable name	ID	description	variance explained (%)
Putative cell type	type	putative cell type defined based on morphological criteria	0,044
Sex	sex	sex of the animal	0,059
Accomodation	acc	ratio of the first AP recorded at 120 pA over the next 3	0,080
time constant (decay)	decay T	time constant of the action potential hyperpolarizing phase	0,129
Resting membrane potential	RMP	membrane potential prior to electrical stimulation	0,148
Action potential onset (time)	APonset	onset of action potential spike	0,173
Action potential threshold	APth	current value preceding the action potential spike	0,178
Hyperpolarization sag	sag	additional inward current during hyperpolarizing current injection	0,215
Rheobase	rheo	current amplitude resulting in pre action potential depolarization	0,226
Minimum frequency	f min	minimum action potential frequency recorded at 120 pA	0,284
Input Tau	input T	time to the cell step response to current injection	0,299
Time to antipeak	t antipeak	time to the negative peak in the action potential time course	0,305
Maximum decay time	t maxD	maximum duration of the action potential hyperpolarizing phase	0,352
Maximum frequency	f max	maximum action potential frequency recorded at 120 pA	0,405
Time constant (rise)	rise T	time constant of the action potential depolarizing phase	0,421
Action potential half amplitude	half ampl	time required by action potentials to reach half peak amplitude	0,421
Action potential peak	peak	peak of positive polarity	0,427
Membrane capacitance	C	membrane capacitance calculated during recordings	0,493
Age	age	age of the animal used	0,522
Maximum rise time	t maxR	maximum duration of the action potential depolarizing phase	0,541
Time to half rise	t halfR	half of the rise time	0,578
Membrane resistance	Rm	membrane resistance calculated during recordings	0,591
Inter event interval	IEI	inter event interval of action potentials recorded at 120 pA	0,600
Event frequency	event f	calculated from average inter event interval	0,633
Instant frequency	instant f	calculated from inter event interval, then averaged	0,644
Cell resistance	rho	resistance of the cell as a conductor	0,646
Action potential half width	half width	action potential width at half-maximal spike amplitude	0,648
Firing threshold	th_current	step current value to elicit action potentials	0,673
Maximum decay value	maxD	maximum value of decay time constant	0,696
Action potential antipeak	antipeak	peak of negative polarity	0,698
Action potential area	area	area under the curve for each action potential recorded at 120 pA	0,743
Time to half decay	t halfD	time to reach half of the hyperpolarizing phase during a spike	0,767
Action potential time to peak	t peak	time for the potential to reach peak value	0,794
Maximum rise value	maxR	maximum value of rise time constant	0,832

Consequently, it was not possible to reduce the total variability to principal components capable of clustering our data based on the first categorization used (type I and II, Fig. 1E). Both type I and type II cells were equally abundant in males and females (male cells N = 14, type I = 6, type II = 8; female cells N = 11, type I = 6, type II = 5), this is consistent with the low contribution of the sex of the animal to the total variance of elecrophysiological properties (Fig. 1F). Instead, a clearer segregation of ACPs was found when data were grouped by age (Fig. 1G).

Overall, this analysis indicates the presence of age-related differences in our recordings. These differences were due to changes in ACP electrophysiological properties (caused by intrinsic or extrinsic factors) rather than the presence of different cell types or sex related differences in our sample. We first assessed the response of ACPs after stimulating the olfactory sensory afferents.

### Postnatal increase in ACP responses to evoked AOB-MC input

**Figure 2:**
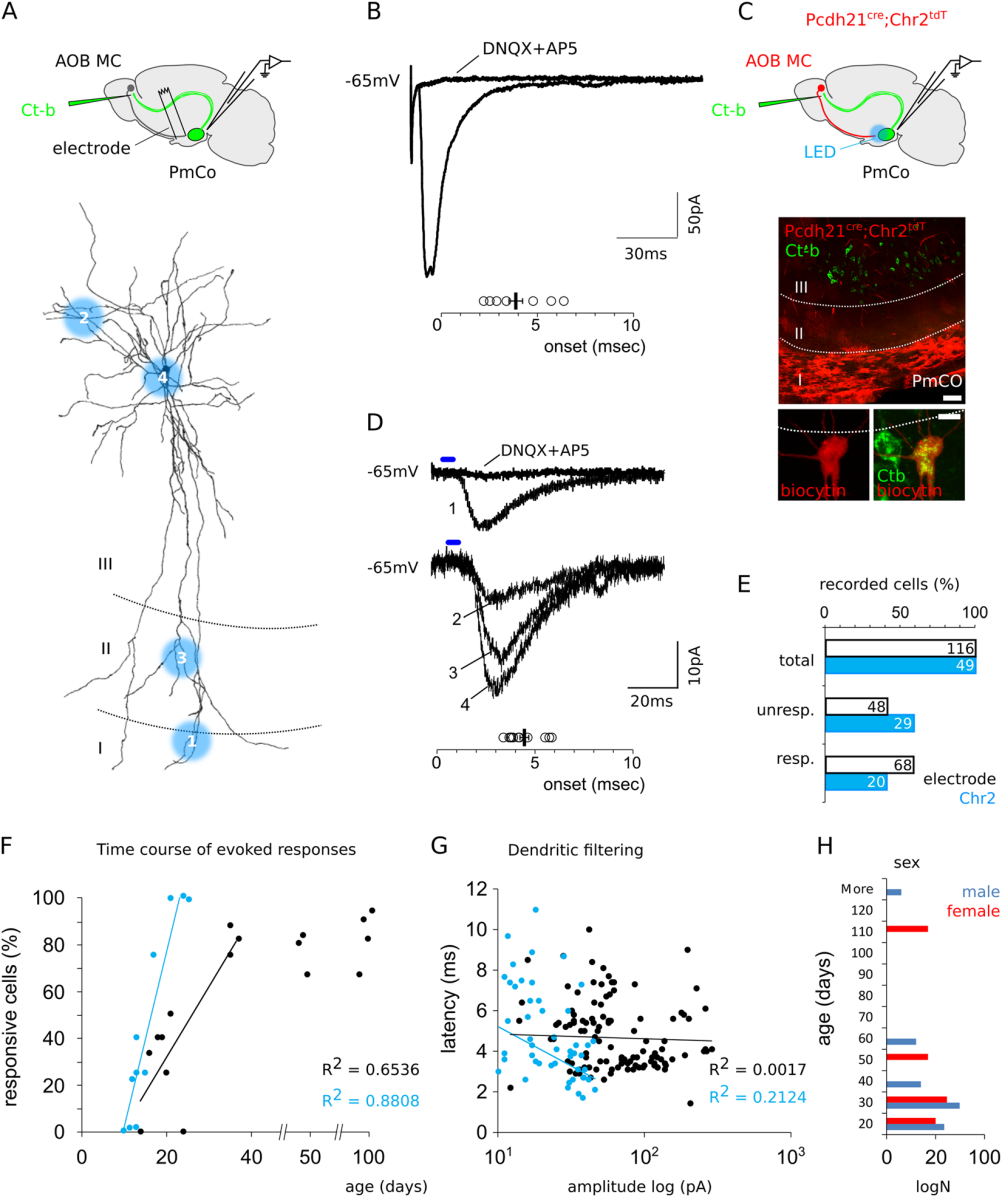
Postnatal increase of evoked excitatory responses in ACPs. (**A**) Amygdala corticobulbar projection neurons (ACPs) were retrogradely labeled via Ct-b (green) injection of the AOB. Incoming input to ACPs are elicited through electrode stimulations of PmCo layer Ia. (**B**) Electrical stimulations of PmCo layer Ia evoke fast excitatory events (onset 3.9 ± 0.6 ms, N = 7) which are blocked with glutamatergic antagonists (DNQX and AP5) in Ct-b-labeled neurons. (**C**) Channelrhodopsin (ChR2) expression was targeted to AOB MCs under the MC-specific *Pcdh21* promoter (in *Pcdh21*^*cre*^*;ChR2*^*tdT*^ mice), allowing optogenetic stimulation of MC afferent axons on different cellular domains of recorded Ct-b-positive neurons. Lower panels: double Ct-b (green)/biocytin (red) labeling was used to identify ACPs in the PmCo and analyze their morphology. (**D**) Excitatory events (onset 4.41 ± 0.2 ms, N = 11) are detectable during light activation of MC afferent fibers in layer Ia of the PmCo but also layer II and layer III (see 1 to 4 numbering on the cell in A), indicating a deep innervation of the PmCo by accessory olfactory afferents. (**E**) Histograms showing the percentages of ACPs responding to electrode (layer Ia) or LED (layer I-III) stimulations. The efficiency rate of both type of stimuli increases over time and reaches approximately 90–100% around 40–50 postnatal days for electrode stimulations (93%) and 25–30 for LED stimuli (100%, **F**). The correlation coefficients are indicated in each plot (LED age range P13-P25; linear fit R^2^ = 0.88; electrical stimuli age range P13 and P37, linear fit R^2^ = 0.6536; later time points are indicated for comparison). (**G**) Scatter plot showing the scarce correlation of EPSC latency with amplitude, as an index of negligible changes in post-synaptic transmission due to dendritic filtering. (**H**) Histogram plot showing the sex composition per age, in the sample (the number of cells is indicated on the x-axis). Scale bars, 50 μm.

The AOB is the main source or sensory input to the PmCo and, therefore, the AOB is a likely source of excitatory input to ACPs^7,10^. However, whether functional synapses exist between ACPs and AOB MCs and whether these are subject to changes during postnatal development, is relatively unexplored. To assess the functional connectivity between AOB MCs and ACPs, we performed patch-clamp recordings of layer II/ layer III ACPs in horizontal slices of the PmCo and measured synaptic input induced by electrical stimulation of layer Ia fibers, which include mainly AOB afferents^10^. ACPs were identified through AOB injections of the neuronal tracer Cholera toxin B (CT-b), which in turn resulted in retrograde labeling in the PmCo (Fig. 2A).

Layer Ia electrical stimulations evoked fast excitatory events (EPSC onset 3.9 ± 0.6 ms, amplitude 103.1 ± 46 pA, N = 7) in Ct-b labeled neurons consistent with a direct synaptic input from AOB MCs (Fig. 2B). To validate these results and obtain a more selective activation of AOB MC fibers, we targeted the expression of the light-gated ChR2 channel specifically to AOB MCs, using a transgenic mouse line in which the floxed ChR2 sequence is activated through Cre activity driven by the MC specific promoter *Pcdh21* (*Pcdh21*^*cre*^*;RChR2*^*tdT*^, Fig. 2C). Optogenetic stimulation of MC axons in all PmCo layers evoked excitatory events in ACPs (EPSC onset 4.41 ± 0.2 ms, amplitude 18.5 ± 2.9 pA, N = 11; Fig. 2D), in accordance with previous reports showing that the PmCo can be innervated by deeper olfactory afferents^10^. The amplitude of responses evoked by electrical stimuli reached significantly higher values compared to those resulting from LED stimuli (Fig. 2G). This difference could imply either that AOB afferents are activated to different extents or that different type of afferents are being activated by electrode and optogenetic stimulations. The partial overlap of response amplitude and the almost complete overlap of the normalized distributions of the responses evoked by both stimuli (Fig. S3A), both suggest this effect might be imputable to a different amount of axons recruited by electrical stimulation compared to LED stimulation.

Finally, both electrode- and LED-evoked inputs were sensitive to blockers of glutamatergic transmission (DNQX, AP5). Together, these results indicate that the PmCo corticobulbar pathway receives direct excitatory inputs from AOB MCs, implying the AOB and the PmCo are reciprocally connected in a closed loop circuit.

We next investigated whether electrically or LED evoked ACP responses change during postnatal development (P10-P100). By delivering current and LED stimulations (current intensity 0.08 mA to elicit reliable spiking, Fig. S1; LED intensity ca. 15 mW, stimulus duration 4 ms, to elicit detectable inward current responses) on ACPs (N = 116 for electrode stimulations, N = 49 for LED stimulations) we found that on average only half of the neurons responded to the delivered stimulus (Fig. 2E). Although this bias could be due to tissue slicing orientation or tissue health, we observed a strong correlation between response percentage and age (P14-P108; Fig. 2F). The age range of the ACPs subject to LED stimuli was chosen to cover the critical stages identified using electrical stimuli (Fig. S4). Furthermore, the absence of any amplitude related variation in response latency in both electrode and LED-evoked EPSCs (Fig. 2G) suggests the absence of changes in neurotransmitter release probability or other effects due to differences in dendritic filtering^11^. Importantly, the increase in evoked responses was observed in both male and female cells (Fig. 2H). These results indicate that ACPs receive direct and increasingly efficient AOB MC input during postnatal development. Given that the most significant changes in evoked responses were observed within the first 2–5 weeks (between P14 and P35), the following analysis is limited to these ages, using 2 and 4 weeks as main time points. Additional time points are added for comparison as needed.

### Conductive properties of ACPs during development

**Figure 3:**
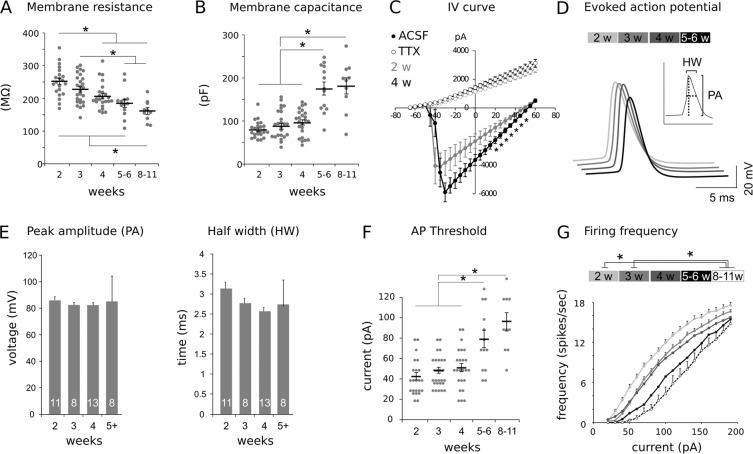
Time course analysis of ACP electrical properties across different developmental stages. (**A**) Comparison of membrane resistance across different developmental stages showed a gradual decrease. Conversely, membrane capacitance followed the opposite trend (**B**). (**C**) Voltage-gated and TTX-sensitive Na+ currents recorded in ACPs through a voltage step protocol (from −65 mV to +65 mV. Voltage step = 5 mV, duration 50 ms). A larger inward Na+ current is seen with a K+ outward current present at positive voltage steps (40–60 mV) in both young (P15, N = 6) and more mature (P30, N = 9) neurons. Differences between 2 and 4 weeks old cells are highlighted by asterisks and limited to a narrow range (20–50 mV) of positive holding potentials. (**D**) Representative AP traces evoked by 120 pA current injections at different ages. (**E**) Age comparison of action potential peak amplitude (PA) or half width (HW). Sample sizes are indicated on the histogram bars. (**F**) Developmental changes in action potential threshold in response to different current injections. (**G**) Decrease in firing frequency induced by different current injections during postnatal development. Data are means ± SEM. See Table 2 for the results of the statistical tests.

Increased responsiveness of ACPs to evoked AOB MC stimulations could be caused by changes in ACP electrical properties. Therefore, ACP membrane resistance and capacitance as well as ACP passive conductive properties at different postnatal ages were examined (Fig. 3A–C).

**Table 2:** Results of the statistical tests used.

Figure	Comparison	Test	Factors	Levels	F value	Sig.	Pairwise comparisons	Sig.
3 A	Membrane resistance	1 w ANOVA	age	5	F_97,4_ = 11.306	p < 0.001	2 vs 4,5–6,8–11 weeks	p < 0.001
3 A							3 vs 5–6,8–11 weeks	p < 0.001
3 A							2,3,4,5–6 vs 8–11 weeks	p < 0.05
3B	Membrane capacitance	1 w ANOVA	age	5	F_97,4_ = 27.604	p < 0.001	2–4 vs 5–6 weeks	p < 0.01
3B							2–4 vs 8–11 weeks	p < 0.01
3 C	IV curve - ACSF	1 w ANOVA	voltage	26	F_25,389_ = 26.72	p < 0.001		
		1 w ANOVA	age	2	F_1,389_ = 25.45	p < 0.001		
		1 w ANOVA	voltage*age	26	F_25,389_ = 2.188	p = 0.001		
		t-test	current*age	26		p < 0.05	20–50 mV (2w vs 4 w)	
3 F	AP threshold	1 w ANOVA	age	5	F_97,4_ = 16.472	p < 0.001	5–6 vs 2,3,4 weeks	p < 0.001
3 F							8–11 vs 2,3,4 weeks	p < 0.001
3 G	Firing frequency	1 w ANOVA	age	5	F_91,4_ = 6.666	p < 0.001	2,3 vs 8–11 weeks	p < 0.05
4 A	EPSCs amplitude	1 w ANOVA	age	3	F_2,46_ = 8.043	p = 0.001	2 vs 4 weeks	p = 0.006
4 A							3 vs 4 weeks	p = 0.003
4B	EPSCs frequency	1 w ANOVA	age	3	F_2,46_ = 6.91	p = 0.002	2 vs 4 weeks	p = 0.004
4B							3 vs 4 weeks	p = 0.026
4 G	IPSCs amplitude	1 w ANOVA	age	3	F_2,19_ = 2.474	p = 0.114		
4 H	IPSCs frequency	1 w ANOVA	age	3	F_2,19_ = 1.876	p = 0.184		
5 C	EPSCs frequency	1 w ANOVA	age	3	F_2,23_ = 6.091	p = 0.008	2 vs 4 weeks	p = 0.0012
5 C							4 vs 4 weeks-AOBX	p = 0.027
6 A	Paired pulse amplitude	2 w ANOVA	peak	2	F_1,779_ = 10.368	p = 0.001		
6 A			age	2	F_1,779_ = 77.109	p = 0.000		
6 A			peak*age		F_1,779_ = 0.462	p = 0.497		
7B	Dendritic length	1 w ANOVA	age	3	F_2,13_ = 6.29	p = 0.015	2–3 vs 4+ weeks	p = 0.014
7D	Spine density	1 w ANOVA	age	3	F_2,13_ = 5.48	p = 0.022	2–3 vs 4+ weeks	p = 0.018
8B	PmCo c-Fos	2 w ANOVA	age	2	F_2,35_ = 9.127	p = 0.005		
8B			odor	3	F_2,35_ = 7.981	p = 0.002		
8B			age*odor		F_2,35_ = 7.247	p = 0.003		
8B	PmCo c-Fos juvenile	1 w ANOVA	odor	3	F_2,15_ = 4.087	p = 0.042	FU vs ctrl	p = 0.036
8B	PmCo c-Fos mature	1 w ANOVA	odor	3	F_2,19_ = 12.328	p < 0.001	MU vs ctrl	p = 0.003
8B							FU vs MU	p = 0.001
8 C	ACP c-Fos	2 w ANOVA	age	2	F_2,26_ = 37.078	p < 0.001		
8 C			odor	3	F_2,26_ = 30.764	p < 0.001		
8 C			age*odor		F_2,26_ = 16.348	p < 0.001		
8 C	ACP c-Fos juvenile	1 w ANOVA	odor	3	F_2,13_ = 5.926	p = 0.018	FU vs ctrl	p = 0.017
8 C	ACP c-Fos mature	1 w ANOVA	odor	3	F_2,12_ = 32.481	p < 0.001	FU vs ctrl	p < 0.001
8 C							MU vs ctrl	p < 0.001
8 C							FU vs MU	p = 0.006
8D	ACP c-fos odor experience	1 w ANOVA	odor	4	F_3,13_ = 40.448	p < 0.001	nMU vs ctrl,fMU,uMU	p < 0.001
8D							uMU vs ctrl	p = 0.005
S2A	Dendritic length	1 w ANOVA	age	3	F_2,104_ = 4.35	p = 0.016	2–3 vs 4+ weeks	p = 0.018
S2A	Dendritic length	2 w ANOVA	distance	9				
S2A			age*distance		F_16,104_ = 1.362	p = 0.18		
S2B	Spine density	1 w ANOVA	distance	9	F_8,104_ = 2.730	p = 0.01	50 vs 150–450 μm	p < 0.01
S2B							100 vs 150–450 μm	p < 0.01
S2B		2 w ANOVA	distance*age		F_16,104_ = 1.478	p = 0.13		
S2C	Branching points	1 w ANOVA	age	3	F_2,104_ = 0.75	p = 0.47		
S2C		2 w ANOVA	distance*age		F_16,104_ = 0.6	p = 0.86		
S3A	Normal distribution	Levene's test			p = 0.002	e		
S3A	Amplitude	Wilcoxon	electrode*LED			p < 0.001		
S4A	Response ratio (electrode)	1 w ANOVA	age (weeks)	4	F_3,15_ = 4.304	p = 0.028	2nd vs 5th	p = 0.039
S4A	Response ratio (LED)	1 w ANOVA	age (weeks)	3	F_2,10_ = 11.925	p = 0.004	2nd vs 3rd	p = 0.032
S4A							3rd vs 4th	p = 0.005
S5A	Amplitude	1 w ANOVA	age (weeks)	3	F_2,58_ = 2.030	p = 0.141		
S5B	Frequency	1 w ANOVA	age (weeks)	3	F_2,57_ = 4.676	p = 0.013	2nd vs 4+	p = 0.023
S5C	Amplitude	1 w ANOVA	age (weeks)	3	F_2,28_ = 2.518	p = 0.1		
S5D	Frequency	1 w ANOVA	age (weeks)	3	F_2,28_ = 2.171	p = 0.134		

While input resistance affects the magnitude of incoming changes in membrane potential, membrane capacitance indicates how quickly a neuron can respond to a change in current. The input resistance of ACPs decreased from ~250 MΩ (2 weeks) to ~170 MΩ (8–11 weeks) during development (Fig. 3A), while membrane capacitance increased from ~90 pF to ~190 pF (Fig. 3B). These findings are suggestive of either changes in neuronal size or ion conductances. However, measures of current-voltage relationships showed no relevant significant changes between 2 and 4 weeks of age (Fig. 3C), indicating that major changes in ACP ion conductivity do not occur within this time window. Therefore, these results imply that differences in the number of responsive ACPs are not due to a shift in intrinsic properties or ion conductances during development.

Because we previously observed developmental differences in ACP firing properties (Fig. 1D), we next evaluated the extent by which ACP conductive properties might affect either cell excitability or evoked currents in current clamp mode. Action potentials were induced by current injection (120 pA) for 600 ms. Membrane potential was clamped at −65 mV by current injection prior to stimulation and single action potentials (APs) were compared across different age groups (Fig. 3D).

AP shape varies across neuronal types and highly depends on ion conductance^12^. AP timing and amplitude both affect firing patterns and, thereby, impact neuronal activity on post-synaptic targets. When comparing AP shape at different developmental stages we did not find obvious differences that would affect neuronal firing patterns in ACPs (Fig. 3D). Neither the amplitude nor the AP half-width changed during the analyzed time frame (Fig. 3E).

The threshold current for AP generation increased over time and peaked after the 4^th^ week of age (5–11 weeks; Fig. 3F). As a consequence, the firing frequency at a given current injection decreased at later stages (Fig. 3G).

Overall these results confirm the absence of major changes in ion conductance altering AP amplitude or time course but indicate a decrease in cell excitability in more mature neurons.

### Analysis of PmCo network properties

Presynaptic factors might also be responsible for altering ACP responses to incoming olfactory input. ACPs receive glutamatergic synaptic input from AOB MCs and other afferents together with inhibitory input from local GABAergic interneurons. Significant developmental changes in this network (e.g. changes in the number of synaptic inputs) would eventually result in changes of spontaneous neurotransmitter release onto post-synaptic ACPs. Spontaneous excitatory (sEPSCs) and inhibitory (sIPSCs) synaptic currents were measured as inward currents (reverse potential around 0 mV) by using different Cl^−^ concentration inside the patch pipette (holding potential of V_H_ = −65 mV) and further distinguished by the application of specific antagonists and/or by a kinetic analysis (see methods).

To explore potential changes in the properties of sEPSCs and sIPSCs in ACPs we analyzed the amplitude and frequency of these events during patch-clamp recordings. We also used the variance/mean ratio of event amplitude to determine whether eventual changes were due to quantal size, release probability or number of release sites^13^, assuming no significant changes in channel properties and numbers. The membrane potential was clamped at −65 mV during the recordings.

**Figure 4:**
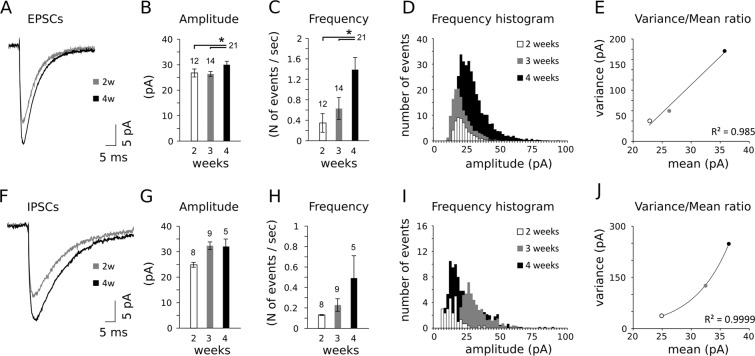
Changes in network spontaneous events across postnatal stages. (**A**) Spontaneous excitatory post-synaptic currents (EPSCs) recorded at P15 and P30. Both EPSCs amplitude (**B**) and frequency (**C**) increase during the peripubertal period. (**D**) Amplitude-frequency distribution of EPSCs in the 3 developmental stages analyzed. (**E**) The analysis of EPSCs mean/variance ratios shows a linear trend in the 3 different stages (R-squared = 0.985), from P14 to P28, which is indicative of an increased number of synaptic contacts. (**F**) Spontaneous inhibitory post-synaptic currents (IPCs) increase in both amplitude (**G**) and frequency (**H**) across the same stages, without reaching statistical significance, as noted in the histogram of amplitude-frequency distribution (**I**). (**J**) The mean variance analysis suggests a change in quantal release of inhibitory synapses impinging onto ACPs prior to the 4^th^ postnatal week (exponential fit), as opposed to release probability (inverse correlation) or number of release site (linear correlation). Sample sizes are indicated above each histogram bar. See Table 2 for the results of the statistical tests.

EPSCs increased both in amplitude and frequency between 2 and 4 weeks of age (Fig. 4A–C). These two effects can be summarized as an increase of excitatory events of larger amplitude occurring at later stages of the postnatal period (Fig. 4D). The shift in synaptic transmission suggests an increased number of synapses, typically resulting in a linear correlation of the variance/mean ratios calculated at different stages (Fig. 4E).

Similar trends were observed in sIPSCs amplitudes and frequency (Fig. 4F–I), but no significant age-dependent difference was found in the amount of ACP inhibition. Although this could be due to sampling related differences (sEPSCs N = 63 cells, sIPSCs N = 22 cells), the comparison of the mean/variance ratios showed a weaker linear correlation (Fig. 4J). To limit the possible bias due to differences in sample size, we have extended this analysis including older ACPs (ca. 10 weeks of age). We found no differences in the amplitude of either EPSCs or IPSCs (Fig. S5A,C), further suggesting that developmental changes in current amplitude are limited or negligible, as already observed at earlier stages (Fig. 4B,G). Conversely, this comparison confirmed the more pronounced increase in the frequency of excitatory and inhibitory inputs during pre- and post-weaning (Fig. S5B,D). The weaker increase in IPSCs frequency could be due to the higher standard deviation in our samples. In fact it would be reasonable to expect an increase in feed-forward inhibition as a consequence of an increased tenure of excitatory inputs on layer I^9^.

The observed increase in EPSCs is coherent with either an increased number of release sites (i.e. synaptic contacts) specifically at excitatory synapses or a heightened activity of ACPs excitatory afferents.

### Quantitative estimate of the AOB afferent input to ACPs

The increased rate of ACP responsiveness to evoked AOB MC inputs led us to hypothesize an increase in their synaptic input onto ACPs. However, since sEPSCs are the results of the activity of AOB MCs in addition to all excitatory synapse activity on ACPs, the observed increase of sEPSCs does not represent a direct proof of changes in afferent synaptic input.

To assess the relative contribution of AOB afferent input specifically, we recorded EPSCs from ACPs (4 weeks of age) after stab-wound lesions of AOB excitatory neurons (AOBX) in juvenile mice (2 weeks old). This lesioning method has been chosen to better circumscribe the damage to AOB MCs, and avoid the damage to MOB projections which could be indirectly connected to the PmCo (via the piriform cortex or other nuclei^14^). Results were compared with EPSCs rates recorded in juveniles (2 weeks old) or more mature (4 weeks old) sham operated animals (sham surgery at 2 weeks of age). In addition, we recorded miniature events (mEPSCs) from ACPs in the presence of the sodium channel blocker TTX (1 µM). The amplitudes and frequency of both sEPSCs and mEPSCs were measured in sham and lesioned mice.

**Figure 5:**
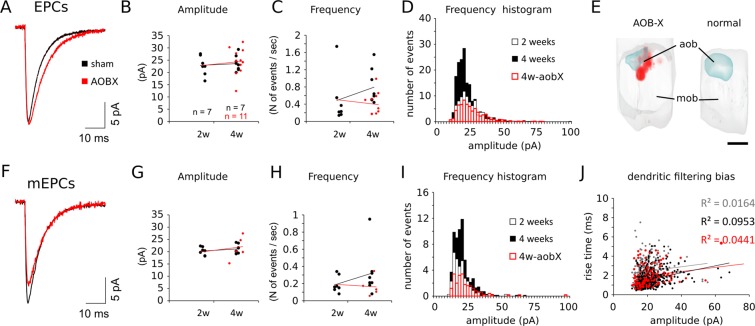
AOB lesion decreases EPSCs and mEPSCs frequency in ACPs. Lesioning the AOB did not affect the amplitude of excitatory postsynaptic currents (EPSCs) recorded from ACPs (**A,B**) but produced a sensible decrease in their frequency (**C**). The amplitude/frequency distribution of EPSCs in 4 weeks old lesioned animals looks similar to 2 weeks old subjects (**C**). (**D**) 3D reconstruction (frontal view) of the main olfactory bulbs (MOB) of a lesioned (AOBX) and a sham operated subject (normal), showing the extent of the lesion in the former and an intact AOB in the latter, for comparison. AOB lesions have similar effects on miniature excitatory post-synaptic currents (mEPSCs, **E**): the average mEPSCs amplitude is unaffected (**F**) while the average frequency is differently distributed and reduced around the mean frequency range (**G**; Chi-square test, cross-tabulation 4 weeks vs 4 weeks-AOBX, df = 180 p < 0.001). (**H**) Scatter plots of the rise times and the amplitude of all mEPSCs recorded in 2-week (grey), 4-week (black) and 4-week-AOBX subjects showing no linear correlations (R-squared values are color coded on the right) indicate the absence of dendritic filtering (e.g. amplitude attenuation in slower events could indicate their distal origin). Scale bar (D), 500 μm. Sample sizes indicated in the first plot are the same for all quantifications. See Table 2 for the results of the statistical tests.

Disruption of AOB output neurons around 2 weeks of age did not result in EPSCs amplitude changes (Fig. 5A,B). However, lesions had an impact on the frequency of sEPSCs preventing it from reaching levels normally observed at 4 weeks (Fig. 5C). A closer look at the EPSCs amplitude frequency distribution showed striking similarities between the values measured in intact 2- and 4-weeks-old lesioned subjects (both lower than sham-operated 4-weeks-old animals; Fig. 5C). The analysis of mEPSCs mirrored these results (Fig. 5E–G). As a result of the elimination of action potential generated events, mEPSCs frequency values were lower, if compared to sEPSCs (Fig. 5C,G) while the amplitude was unchanged overall (Fig. 5B,F). AOB lesions reduced mEPSCs frequency values to 2 weeks levels, in 4 weeks-AOBX subjects (Fig. 5G). The change in mEPSCs frequency was independent of dendritic filtering, as the rise times and amplitudes were uncorrelated at all the time points considered (Fig. 5H).

These experiments indicated that AOB afferents contribute to more than half of the excitatory events recorded from ACPs at the end of the 4^th^ week of age. In addition, they indicate that changes in sEPSCs frequency are likely to reflect direct changes in the amount of functional AOB MC-to-ACP synapses.

### Release probability at the AOB MC to ACP synapse

Because changes in release probability and quantal release can contribute to the increased rate of evoked ACP responses, we measured paired pulse ratios (or PPR^11,15^) and estimated the readily releasable pool of synaptic vesicles at the MC to ACP synapse.

**Figure 6:**
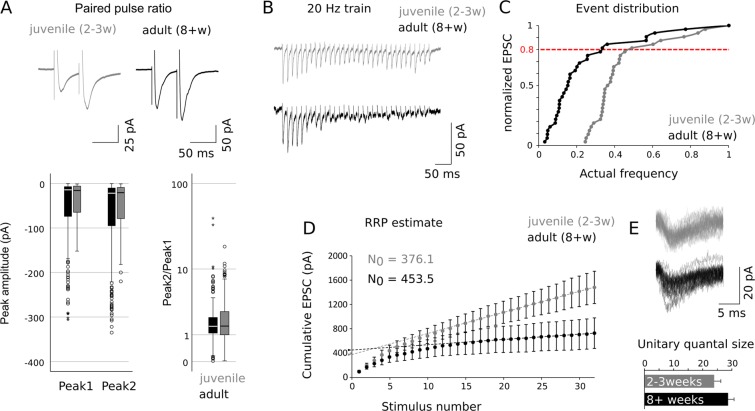
Estimates of release probability and vesicle pool size at the MC to ACP synapse. (**A**) Representative examples of ACP responses to paired electrical pulses delivered to PmCo layer Ia. The average peak amplitudes are shown in the box plots on the bottom left. Peak ratios (bottom right) are calculated as the amplitude of the second peak over the first peak. (**B**) Representative traces from mature (black) and juvenile (gray) ACPs in response to stimulation of layer Ia of the PmCo for 2 s at 20 Hz. (**C**) The train stimuli used to extrapolate RRP size values (from 26th to 33rd) were obtained from cumulative frequency distributions of normalized EPSCS amplitudes by setting a threshold of 0.8 cumulative release probability. (**D**) Cumulative amplitude plots of population averages for juvenile (gray, n = 6) and adult (black, n = 5) ACPs. Linear fits to the final 7 pulses responses are extrapolated back to the y-intercept. Intercepts (arrows) indicate estimate of current from readily releasable vesicles. (**E**) Representative mEPSC traces recorded from juvenile and mature ACPs were used to estimate the unitary quantal size (N = 3 per group). Data are expressed as mean ± SEM. See Table 2 for the results of the statistical tests.

To this aim we delivered electrical stimulations with inter-stimulus intervals of 50 ms (Fig. 6A) to layer Ia fibers of the PmCo and then quantified the paired-pulse ratio of peak amplitudes (EPSC2/EPSC1) in juvenile (2–3 weeks old) and adult (8 weeks or more) subjects. The amplitudes of evoked responses recorded from ACPs in mature animals were significantly larger compared to those of younger subjects (2–3 and 8+ weeks). However no significant interaction was found between age and ratio (Fig. 6A). The PPR was not significantly different in adult animals compared to juveniles (Fig. 6A) and no interaction was found between the two factors (age and peak amplitude), indicating the presence of paired pulse facilitation (PPF) at both ages.

Another method to quantify the extent of neurotransmitter release at a given synapse implies an estimate of the vesicle recycling rate, measuring the so called readily releasable pool (or RRP) by stimulating a synaptic terminal with a high-frequency train of action potentials^16^.

Trains of 33 electric pulses were delivered at 20 Hz and 0.8 mA, a supra-threshold current intensity for ACPs (Fig. 6B,C). RRP size was estimated by plotting the cumulative EPSC amplitude as a function of stimulus number (Fig. 6D).

RRP size estimates were obtained by intercepting linear fits to the last 7 points of the cumulative EPSC curve with the y-axis of such plot (Fig. 6C). An average RRP size of 453 pA was estimated for adult (P150) and 376 pA for juvenile (P18) ACPs (Fig. 6D). These values were divided by the average mEPSC amplitude to obtain an estimate of the unitary quantal size^15^ (Fig. 6E). Since this parameter did not differ significantly, we conclude that RRP differences can be explained by an increase in the total amount of releasable vesicles. Taken together these results indicate that increased ACP responses to AOB MC afferent stimulation are likely not caused by an increase in either release probability or quantal sizes, further supporting the idea of a change in the number of AOB MC-to-ACP synapses during development.

### ACP morphological development

**Figure 7:**
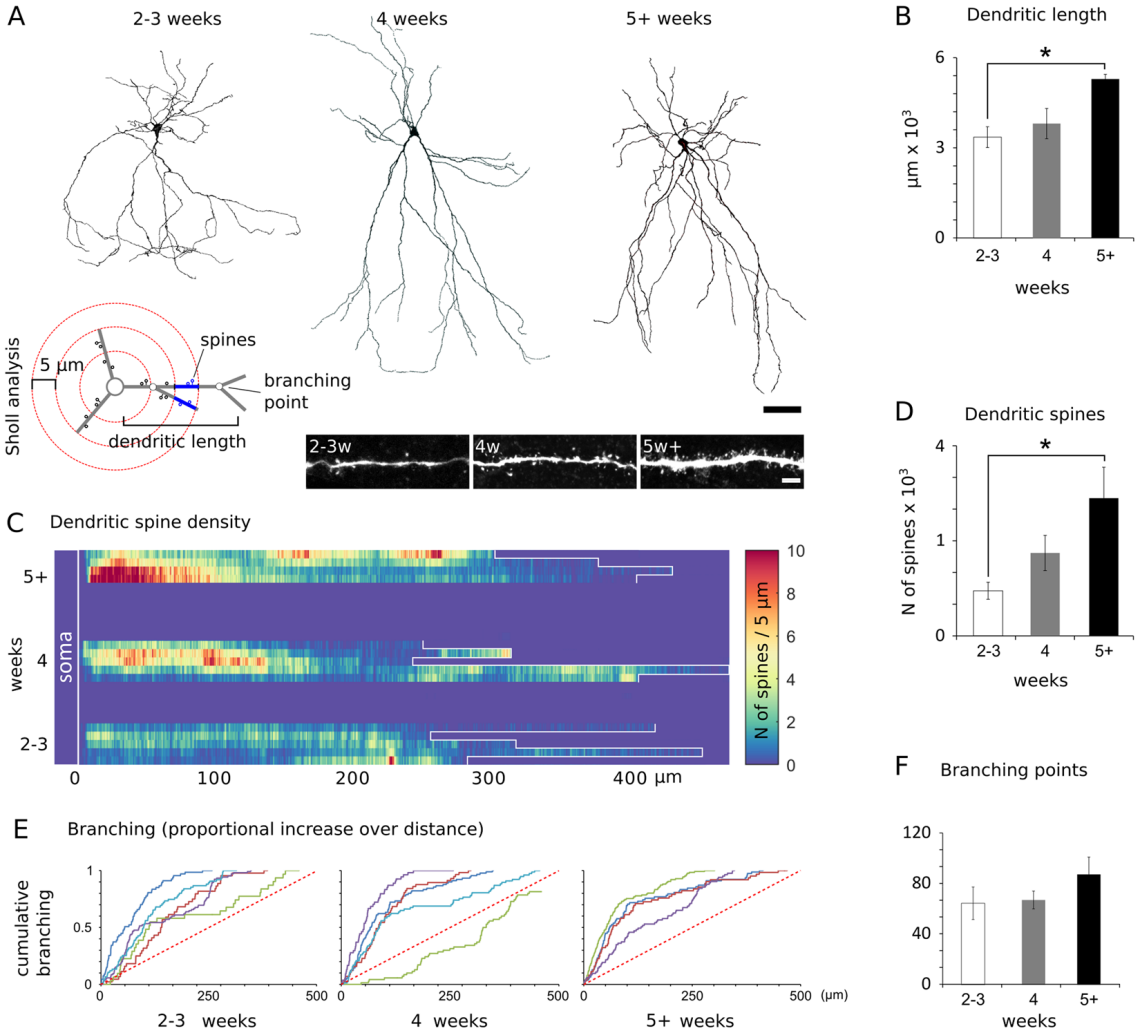
Morphological changes of ACP during postnatal development. (**A**) Sampled biocytin filled ACP at different developmental stages (in weeks). The diagram on the left summarizes the parameters analyzed using the Sholl analysis. Measures of each parameter are referred to all neuronal processes detected at each radial distance considered (blue segment in the diagram). The panels on the right show representative samples of proximal and distal dendritic branches at different developmental stages. (**B**) Comparison of dendritic lengths at different ages (N = 5 cells each). (**C**) Density plot showing the dendritic spine distribution obtained from the Sholl analysis in ACPs sampled at different ages (2–3 weeks, N = 5; 4 weeks, N = 5, 5+ weeks, N = 4). Dendritic spine densities (**D**, N = 5 cells each) and branching points (**E,F**) at different ages. (**E**) Cumulative branching curves obtained from the analyzed neurons show no drastic age-dependent changes (**F**) in dendritic organization and indicate homogeneity in the sampled neuronal morphologies. Scale bars 50 μm (**A**), 25 μm (**C**). See Table 2 for the results of the statistical tests.

Since AOB lesions decreased the frequency of post-synaptic excitatory currents recorded from ACPs by about 50% (Fig. 5C), a postnatal increase in the amount of synaptic inputs from the AOB might eventually result in detectable changes in ACP dendritic spine density. The observed changes in ACP passive and active electrical properties (membrane resistance and capacitance, Fig. 3), are already suggestive of sensitive developmental changes in neuronal morphology. To assess and measure the extent of ACP morphological changes and quantify the dendritic spine density on ACPS, we analyzed a sample of 25 neurons filled with biocytin during our recordings (filling time ca. 15–20 minutes per cell; Fig. 7A). We then used Sholl analysis to estimate the amount of spines on all reconstructed neurons (step 5 μm, Fig. 7A).

This survey revealed a general increase in dendritic length (Fig. 7B), which was more pronounced in the proximal dendritic domains (Fig. S2A). The density of dendritic spines was also age dependent without being localized on specific cellular domains (Figs. 7C,D and S2B).

Overall, the total number or spines increased with age, with a peak spine density at 5 weeks and after (ages considered ca. P40-P100). We also evaluated the degree of dendritic branching as a measure of morphological complexity (Fig. 7E). This analysis revealed a more dramatic age dependent increase in the complexity of proximal dendritic arbors (Fig. S2C) but did not reveal any clear effect of age in this regard (Fig. 7F).

These results indicate that ACPs undergo drastic morphological changes during postnatal development, in terms of neuronal size, spine density and dendritic complexity. Changes in dendritic spine density are more pronounced – with an almost 2-fold increase at each developmental stage considered (Fig. 7D). These results are compatible with a general increase in afferent inputs, including those originating in the AOB.

### ACPs show increased responses to social odors

The increase in evoked responses to AOB MC input suggests ACPs might be activated by olfactory input with increasingly higher probabilities during their maturation (from P20 to ca. P40; Fig. 1).

**Figure 8:**
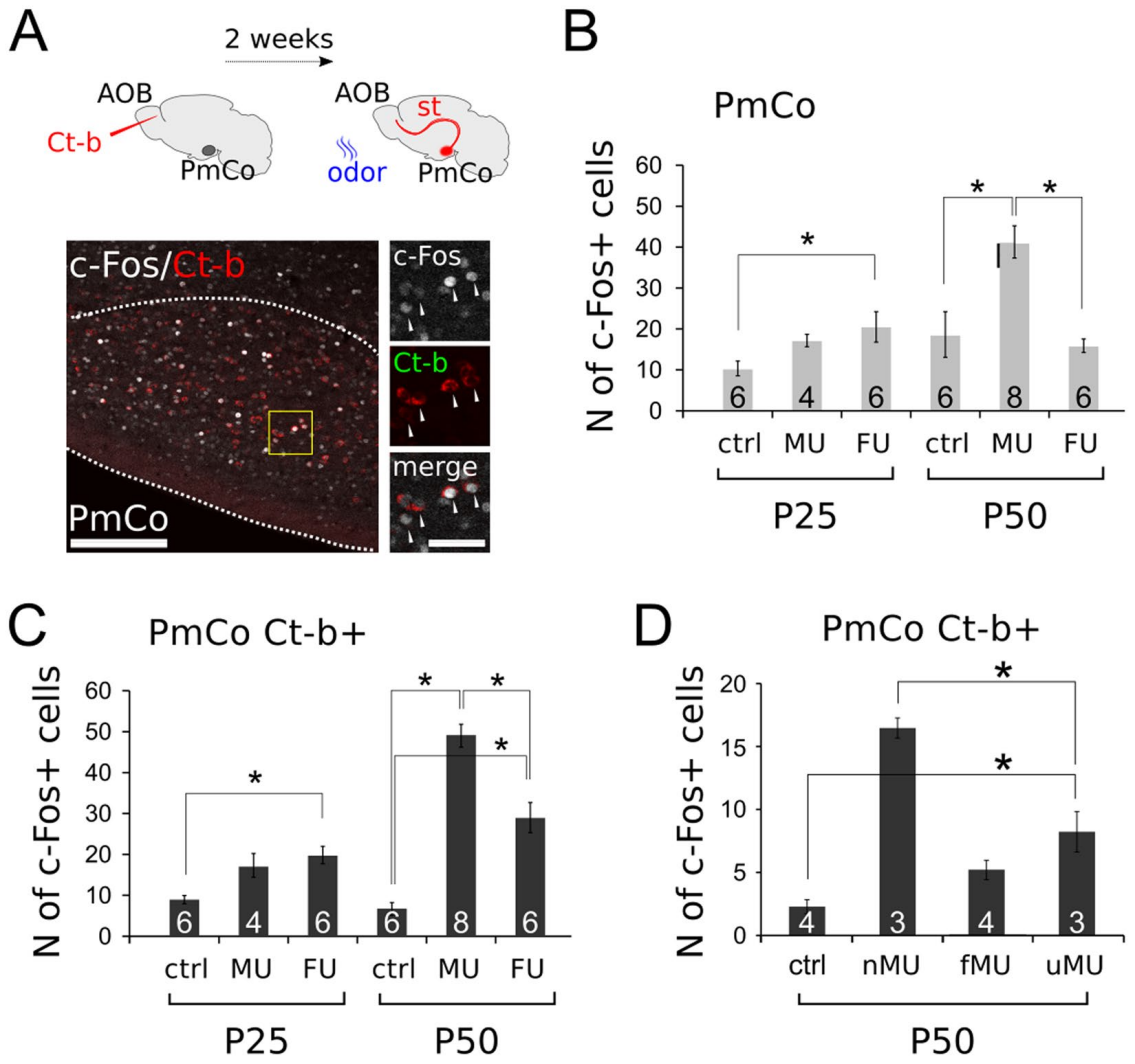
Responses of PmCo corticobulbar neurons to urine odor stimuli. (**A**) Sagittal section of the PmCo showing c-Fos/Ct-b colabeling (insets are magnified from the region highlighted in yellow). (**B**) Comparison of c-Fos expression in the whole PmCo after exposure to home-cage odors (ctrl), male (MU) or female (FU) urine stimuli in juvenile (P25) and mature (P50) female mice. Male odors do not activate significantly PmCo neurons in juvenile females, but female odors do. Conversely, male odors activate PmCo neurons more than female odors in mature female mice. (**C**) Quantification of c-Fos expression in Ct-b-positive PmCO neurons (ACPs) in response to the same odor sources, in the same mice. ACPs do not show sex-specific responses in juvenile female mice. ACPs show sex specific odor responses in mature female mice. (**D**) Effect of mature (P50) experience in ACP odor responses. Exposure to homecage odors is compared to novel (nMU), familiar (fMU) and unfamiliar (uMU) odors. Size of each sample is indicated on the relative histogram bar. Data are expressed as mean ± SEM. Scale bars 50 μm, 200 μm. See Table 2 for the results of the statistical tests.

To address this point, we investigated the colocalization of the activity marker c-Fos in Ct-b positive PmCo ACPs after olfactory stimulation of 3- and 7-weeks-old mice (Fig. 8A). Since female mouse responses to male odors have been well characterized both in juvenile and mature animals^17^, female mice were used in this analysis.

To identify PmCo ACPs, Ct-b was injected rostral to the AOB and allowed to diffuse towards the AOB GC layer (see methods^7^). Female mice at the age of P25 and P50 were exposed for 30 minutes to urine-derived odors obtained from unfamiliar male or female mice. Quantitative analyses of c-Fos expression in the whole PmCo showed that odor exposures had almost opposite effects in juvenile versus mature females. In younger females, female urine odors elicited significantly higher c-Fos expression when compared to unstimulated subjects. Conversely, in mature females, male urine odors induced higher c-Fos expression compared to female derived cues or unstimulated controls. (Fig. 8B). A similar shift in activated cells was observed in Ct-b labeled ACPs (Fig. 8C). Interestingly, the time window defined by this change coincides with the age at which mice begin to display a higher motivation to investigate opposite-sex odor sources and develop an amygdala-dependent attraction which is unaffected by hormonal modulation but relies instead on repeated experience^17,18^. Thus, if changes in ACP innervation by AOB MCs or odor evoked activation are a consequence of the value attributed to social odors, attractive/experienced odors would likely activate ACPs more effectively and induce higher c-Fos expression levels.

To address this question, we analyzed c-Fos expression in ACPs of mature female mice after exposure to either novel (nMU) or experienced male urine stimuli (experienced or familiar male stimuli consisted in bedding material female mice have been previously exposed to for a week, fMU). A third group of females was exposed to novel odors, after an initial 1 week exposure to a different set of male odors (unfamiliar male urine stimuli, uMU).

In P50 females, novel odor stimuli elicited the highest c-Fos expression levels (Fig. 8d). Conversely, exposure of female mice to familiar-experienced odors, which typically produces a selective attraction, did not induce comparable levels of cellular activation. Unfamiliar odors induced intermediate levels of c-Fos expression, compared to unstimulated controls and females exposed to novel stimuli (Fig. 8D), suggesting that ACP response to novel stimuli might be partially affected by odor experience.

Overall these results indicate that both the PmCo as a whole as well as its corticofugal circuits are significantly more active in response to odor stimulation after juvenile stages (towards sexual maturity). The functional activation of ACPs does not appear to be dependent on the associative processing of odor value occurring during olfactory familiarization. It is possible that, once functionally mature, ACPs may enhance the tuning properties of the AOS at a stage in which the processing of sensory inputs becomes more complex and requires more sensitivity.

## Discussion

In the present study, we show that evoked input from AOB mitral cells (MCs) becomes increasingly efficient in the activation of amygdala corticofugal projection neurons (ACPs). Using optogenetic stimulations, we demonstrate that the strength of MC inputs to ACP basal dendrites at superficial and deeper levels of PmCo layer I is age-dependent. Whereas analysis of ACP electrophysiological features revealed no obvious contribution to the circuit maturation. Instead, since the tenure of ACP spontaneous excitation – normally increasing between the 2^nd^ and 4^th^ postnatal week - remains low after AOB lesion, we hypothesized that a large amount of the presynaptic excitatory inputs to ACPs originates from AOB MCs during this time window (2 weeks). Therefore, both EPSC mean-variance analysis and estimates of dendritic spine density at different stages, are coherent with an increase in the amount synaptic inputs to ACP, while other quantal parameters of synaptic transmission remain unaltered after the 2^nd^ postnatal week. The higher level of c-Fos expression induced by odor exposure in both the PmCo and ACPs after this developmental period, further supports that cortical feedback neurons from the olfactory amygdala are more efficiently recruited by olfactory stimuli after juvenile stages.

### Delayed wiring of mitral cells to downstream targets

Through electrical and optogenetic stimulations of afferent inputs to ACPs, we show a gradual increase of evoked responses, which can be attributed to an increase in the amount of active synaptic sites. However, changes occurring on the presynaptic side of the activated synapses may also contribute to this result. In particular, since the increased ACP responsiveness is measured stimulating AOB MC axons and in general the ACP rates of spontaneous excitation, the extent by which afferent circuits are active or able to conduct electric signals should be considered as possible causes.

Although MC axons reach peak conductivity shortly after P15^19^, this does not rule out that MC could get increasingly more active after this stage. However, MC firing frequency does not appear to have a similarly linear maturation, except for the firing patterns becoming more irregular^20^. Moreover, the glomerular wiring of MC apical dendrites is also accomplished by the 2^nd^ postnatal week^21^, indicating that overall MC input and output connectivity has reached a mature stage by 2 weeks of age. In addition, although differences might exist between MOB and AOB MCs^22^, AOB MC electrophysiological properties seem to be rather stable between P14 and more mature stages^23^. Taken together, these considerations suggest the absence of sensible changes in the conductance of MC axons across the ages tested, although their morphological development may extend to later stages.

Although MC genesis begins earlier than the rest of the local OB circuitry^24^ (embryonic days 11–13), MCs finalize their development postnatally around P10^18^. This implies that at the time of our recordings (2^nd^ to 4^th^-5^th^ postnatal weeks), MCs had already completed most of their electrophysiological and morphological maturation. However, substantial reorganization of MC dendritic arbors and their axonal efferents continues into later stages^25^ and morphological analysis of the lateral olfactory tract (LOT, i.e. the fiber bundle formed by MC axons) shows that the innervation of olfactory nuclei (downstream of the MOB) proceeds at least until the first 2 postnatal weeks^23^.

 Despite AOB neuronal development and layering seemed to be similarly paced^23^, with MCs being generated even earlier than in the MOB^23^, the thickness of the AOB LOT continue to increase until the 2^nd^-4^th^ postnatal weeks (P15^23^; P30^26^) and maintains growth potential until adult stages, indicating that AOB MCs continue to grow their axons beyond the juvenile period. Finally, olfactory bulb lesions carried out at different postnatal days (between 0 and 33) result in MC axonal degeneration in the cortical amygdala only after the second postnatal week, later than other higher order olfactory cortices^27^. Thus indicating that innervation of the rodent cortical amygdala by olfactory afferents occurs for the most part after 2 weeks of age. Overall, these considerations support the likelihood that changes in ACP evoked responses can be explained by rearrangements of the afferent synaptic contacts established by AOB MCs.

### Selective activation of AOB MC axons

In our study we have used different methods to activate MC axons (electrode and optogenetic stimuli) while recording from PmCo feedback projection neurons. Although we obtained similar results using both methods (Figs. 2, S3 and S4), neither of the two is obviously comparable to physiological stimuli. Thus, the time course outlined in our stimulation experiments might not precisely reflect the maturation of MC-ACP synapses to inputs from the olfactory bulb. Despite this technical drawback, the time window we broadly identified using electrode stimulations (2^nd^ to 4^th^-5^th^ postnatal weeks) is compatible with the results of the rest of our experiments (LED evoked responses, sEPSCs, AOBX, c-Fos). Future *in-vivo* experiments will be required to confirm these findings.

Additional concerns might arise by comparing electrode to LED-evoked responses due to their significant amplitude differences (responses to LED stimuli are smaller; Fig. 2B,D). In fact, this difference could suggest the activation of different pathways by the two types of stimulations. However, the following considerations could rule out this possibility. First, currents evoked by electrode and LED stimuli show partially overlapping amplitude distributions (Fig. S3A,B), suggesting that amplitude differences might be mainly due to the higher variability of electrode-evoked responses. Second, while electrode stimulations were set at ACP firing threshold levels, the power of LED stimuli was set to elicit detectable inward current responses, which are likely lower than those required to elicit action potentials. This has been done to limit the effects of light stimulations to the area in which the recorded cell was located (see methods). Such intensity may not be sufficient to stimulate the same amount of axons activated instead during electrode stimulations, but was chosen to avoid the potential excitation of MC axons (originated either in the AOB or in the MOB) targeting other neurons projecting to ACPs (for example from the piriform cortex^14^). Third, because of the decreased density of olfactory afferents across different PmCo layers (decreasing drastically from layer I to layer II-III^10^, where ACPs are localized), a lower number of MC axons can be targeted by LED stimulations. Incidentally, electrically evoked responses in ACPs were triggered using electrodes with a diameter of approximately 150 μm, which likely activated a higher number of MC axons, compared to LED stimuli. Lastly, in general, even using the highest LED intensity, it may not be possible to induce responses of amplitude comparable to electrically evoked stimuli due to the extent of ChR2 expression achievable with a single generic inducible ChR2 knock-in allele is often sub-optimal even in the same neuronal type^28^.

Nonetheless, it is quite possible that the synapses activated on ACP basal dendrites (layer Ia) by electrode stimulations are different in nature compared to those probed by LED stimuli on other cellular domains. Not only could these synapses could be different in nature but also they could be related to a different type of afferent neurons. A finer anatomical and electrophysiological characterization of AOB inputs on ACPs will be required to resolve these confounds.

### Other factors possibly explaining changes in ACP responses to evoked AOB input

Despite the decreasing responsiveness of ACP to intracellular current injections with age (Fig. 3G), post-synaptic mechanisms dampening AOB-ACP synaptic transmission and resulting in an increase in the response rate to evoked AOB MC stimuli may exist. In fact, it is possible that a gradual development of different modulatory systems (involving for example catecholamines^3,29,30^, hormones^31^ or endogenous opioids^32,33^) affected circuit wiring and the rate of synaptic development at the post-synaptic side of the MC-ACP synapse.

Nonetheless, the following considerations suggest a minor role of post-synaptic mechanisms in explaining our results: (1) Modifications of AOB input (through lesion) are enough to cause significant changes in detectable excitatory inputs recorded from ACPs (i.e. an increase in AOB synaptic input would be detectable regardless of other factors). (2) The amplitude of individual EPSCs and mEPSCs does not change significantly during development, which suggests that individual synaptic events are not significantly amplified by any post-synaptic modulators. (3) Cellular inward and outward currents affected by age (i.e. no drastic changes in ion channel conductivity occur). (4) Blood hormone levels are relatively stable at most of the time points considered; hormones peak either slightly after birth or around the completion of pubertal maturation (around 8 weeks of age^34^) but they do not increase in a gradual continuous fashion, as do ACP responses to AOB input. (5) The expression of hormone receptors follows a similar trend in the PmCo (i.e. stable levels during juvenile development^35^), ultimately suggesting a marginal role of circulating hormones. (6) We have tried to maintain an equal sampling of male and female cells in our electrophysiology experiments and we haven’t found any evidence of sex-related differences (potentially caused by differences in circulating sex hormones). However, since we cannot exclude other indirect effects – e.g. hormone-mediated gene regulation – future studies will be necessary to carry out more conclusive assessments.

In addition to post-synaptic modulatory factors, ACP responsiveness to evoked AOB input could be heightened through parallel excitatory (or disinhibitory) circuits.

We tend to exclude this possibility for the following reasons: (1) No indirect ACP excitation was observed during stimulation of PmCo layer Ia, indicating that eventual parallel afferents to ACPs and activated by AOB MCs are not coursing through it (these could include for instance (considering 300 μm thick slices): medial posterodorsal and posteroventral MePd, MePv, posterolateral cortical PlCo, basolateral BLA, lateral LA, central CeA between Bregma −1.82 and −2.18, PlCo, basomedial BMA, LA, entorhinal cortex Ent, cornus ammonis area 3 (CA3) between Bregma −2.30 and −2.80; (2) Optogenetic activation of ChR2 expressed in MC afferents never induced detectable responses when LED stimuli were delivered far from ACPs (i.e. somata, apical or basal dendrites) and in proximity of nearby amygdala nuclei or the piriform cortex, even when intermediate and maximum LED intensities were used (50% and 100%, see methods).

## Conclusions

Our results, together with previous findings, delineate at least 3 distinct phases in the functional development of the olfactory amygdala. (1) During the early postnatal phase (1^st^ and 2^nd^ weeks) mice display strong (aversive or attractive) responses to external stimuli, either mediated innately or by maternal influence^1,2^. (2) The juvenile phase (3^rd^ and 4^th^ weeks, prepubertal stage) is characterized by experience-dependent odor learning through the early involvement of amygdala circuits^3^; (3) Adolescence (5^th^ week onwards) coincides with behavioral changes associated to richer and more diverse olfactory experiences, sanctioning the onset of a more complex processing of social signals through the amygdala^17^.

Importantly, it is only during adolescence that mice begin to disperse out of their nest^36^ and thus start to require better sensory capabilities to discern, for instance, related vs. unrelated individuals, suitable partners and potentially harmful odor sources. Conversely, during the first two developmental stages (P0-P14 and P15-P30), the pressure for a fine odor tuning within the AOS might be lower. Therefore, the gradual development of feedback circuits originating in the accessory olfactory amygdala, could reflect the need to adapt to changes in the complexity of the sensory environment by adjusting the accuracy of its neural representations.

## Methods

### Animals

Mice were housed in the temperature- and light-controlled Children's National Medical Center animal care facility and given food and water *ad libitum*. All animal procedures were approved by Children's National Medical Center's Institutional Animal Care and Utilization Committee (IACUC) and conformed to NIH Guidelines for animal use. *Pcdh21*^*cre*^ animals were kindly donated by Dr. Kevin Briggman (Tg(Cdhr1-cre)KG76Gsat; RRID:MMRRC_036074-UCD), and ChR2tdT mice were obtained from Jackson Laboratories (Ai27D or Ai27(RCL-hChR2(H134R)/tdT)-D).

### Stereotaxic injections and lesions

Cholera toxin subunit-B (Thermo Fisher Scientific; Alexa Fluor® 555 Conjugate, C34776; Alexa Fluor® 488 Conjugate, C22841) was diluted to 10 μg/μl in sterile PBS, aliquoted and stored at 4 °C until use. Injection of tracers were as follows. Mice (age span postnatal day 10–100) were anesthetized by IP injection of 10 μl/g body weight anesthetic cocktail containing 10 mg/ml ketamine and 1 mg/ml xylazine prepared in sterile saline solution. Bregma coordinates of injection sites targeting the AOB granule layer were X: −2 mm, Y: 0.9 mm, Z: −2.6 mm and with a 45° angle. In younger animals, coordinates were adjusted accordingly. Injections (50–100 nl) were made bilaterally using beveled glass pipettes (Kingston Glass) at depths of 5.1–5.3 mm from the pial surface. Tracer injections were manually assisted using a Pico Injector (Harvard Apparatus, pli-100), with each pressure step delivering approximately 10–20 nl per minute. Ten minutes after the last injection the glass pipettes were withdrawn and the wound was sutured. Stab wound lesions targeting the AOB mitral cell layer were performed following similar procedures (Bregma Z: −2.4) by lowering a blunt syringe needle (100 Sterican, 0.4 mm diameter × 25 mm length, Ref# 9180117, Braun) filled with saline solution into the AOB mitral cell layer (approximately 1 mm length, 0.5 mm wide, 0.5 mm deep in adult mice). Lesions were caused merely by mechanical damage. In order to make sure the lesion would span the majority of the AOB mitral cell layer, the needle was repeatedly withdrawn and lowered into adjacent positions along the rostral to caudal axis. Visualization of AOB MC lesions were obtained histologically after collecting the brain tissue to perform ACP recordings from Ct-b positive neurons in the PmCo.

### Immunohistochemistry

Mice were anesthetized with a 100 μl 4:1 cocktail of ketamine (100 mg/ml) and xylazine (20 mg/ml) and perfused transcardially with 0.9% saline solution followed by 0.1 M phosphate buffer (PB) containing ice-cold 4% paraformaldehyde. The brains were removed, post-fixed overnight in 4% paraformaldehyde, then incubated for 48 h in 0.1 M PB containing 30% sucrose, and embedded in OCT. Cryosections (30 μm thick) were collected on a cryostat and mounted on SuperFrost Plus glass slides (Fisher Scientific) for immunofluorescence analysis. Tissue sections were washed for 10 min in PBS, incubated for 1 h at room temperature in blocking solution (0.5% Triton X-100, 2% donkey serum in PBS) and incubated overnight at 4 °C in blocking solution containing the primary c-Fos antibody (1:500, rabbit, #sc-52, Santa Cruz; RRID:AB_2106783). Tissue was washed for 10 min in PBS, followed by incubation in secondary antibody for 1 h at room temperature (1:1000, Alexa fluor 647 conjugated donkey anti-rabbit; RRID:AB_2536183).

### Acute brain slice preparation

Acute slices were prepared from 2–4-months-old male and female mice. Animals were briefly anesthetized with CO_2_ and decapitated. Brains were removed quickly and placed in cold (~10 °C), sucrose-based oxygenated (95% O_2_–5% CO_2_) cutting solution composed of (in mM) sucrose 234, glucose 11, NaHCO_3_ 26, KCl 2.5, NaH_2_PO_4_ H_2_O 1.25, MgSO_4_*7H_2_O 10, and CaCl_2_ H_2_O 0.5. Coronal slices containing the PmCo were obtained with a slicing vibratome (VT1200s; Leica, Wetzlar, Germany). The brains were trimmed by removing the cerebellum with a perpendicular cut to the rostral–caudal plane and the caudal brain surface was glued (Superglue Loctite 401 20G, Loctite) to the vibratome stage submerged in cold cutting solution. Slice thickness was 300 μm for all experiments. The slices were immersed in oxygenated (95% O_2_/5% CO_2_) artificial cerebral spinal fluid (ACSF) at 34 °C for 30–45 min. ACSF was composed of (in mM) NaCl 126, NaHCO_3_ 26, glucose 10, KCl 2.5, NaH_2_PO_4_ H_2_O 1.25, MgCl_2_*6H_2_O 2, and CaCl_2_*2H_2_O 2, pH 7.4, with osmolarity maintained at 290–300 mOsm.

### Slice electrophysiology

Slices were transferred, to a recording chamber and superfused with ACSF. All experiments were conducted at room temperature (22–24 °C). Patch-clamp recordings were performed using an upright microscope (Nikon E600 F, Tokyo, Japan), equipped with ×10 and ×60 objectives and DIC optics. Neuron types were identified by their morphology, intrinsic properties, and layering within the different nuclei examined (OB or PmCo). In some recordings biocytin (3–5%, B1592, Thermo Fisher Scientific) was added to the intracellular solution containing (in mM) 130 K-gluconate, 10 NaCl, 10 HEPES, 0.6 EGTA, 2 Na_2_ATP, 0.3 Na_3_GTP. In some cases, when inhibitory currents were recorded a high chloride solution was used containing (in mM): 70 K-gluconate, 70 KCl, 10 HEPES, 10 EGTA, 2 MgCl_2_, 2 Na_2_ATP, 0.3 Na_3_GTP. To block AMPA and NMDA receptors, 20 μM DNQX and 100 μM DL-AP5 (Tocris Bioscience), respectively, were added to the ACSF. Inhibitory currents were usually blocked with bicuculline 10 μM (Tocris Bioscience).

Recordings were made using a Multiclamp 700B amplifier (Molecular Devices) digitized at 10–20 kHz and acquired using Clampex Software (pClamp 10, Molecular Devices). For most recordings, pipette resistance was 3–6 MΩ. Series resistance was normally <30 MΩ and periodically monitored. Bessel was set at 1 kHz for all voltage clamp and 10 kHz for current clamp experiments. Gain was set at 5 V/V in current clamp recordings. A patterned LED light illuminator (Polygon 400, Mightex, model # DSI-E-0470-000) was used to illuminate tissue sections during experiments involving optogenetics (Light source 470 nm, ca. 1–5 mW, Mightex).

LED intensity was set at a fixed level, such that its effects were limited to the camera field of view, allowing targeted stimulation of different PmCo cellular layers. Full-field illumination was used unless stated otherwise, with the LED intensity set at approximately 10% of the maximum using a CFI Fluor 60XW objective (Nikon). The estimated field-of-view was approximately 0.39 mm ×0.695 mm, for an estimated output intensity of 498 mW/mm^2^ (or ~15 mW for the objective field-of-vew of 0.13 mm ×0.23 mm), which gave us the best control of LED spatial specificity. Full-field illumination did not alter the amplitude of light-evoked responses. Electrode stimulation of PmCo layer Ia was carried out using platinum iridium electrodes (125 μm, FHC Neural Microtargeting Worldwide #30025). Layer Ia and the adjacent layer Ib were distinguished using anatomical and morphological criteria (tissue texture, relative position and absence of cell bodies). Individual EPSCs latency was defined by a change in inward current amplitude corresponding to more than twice the baseline noise standard deviation, measured after the detectable stimulation artifacts (induced by either electrode or LED-derived stimuli).

### Paired pulse ratio and RRP estimates

For both paired pulse and train stimuli a supra-threshold current initiated evoked responses in ACPs (Fig. S1). Paired pulse stimulations followed previously established protocols^11,15^. Briefly, pairs of electric pulses were repeated at 0.5 Hz (50 ms inter-pulse interval) and averaged over 30 consecutive trials. The ratio of the average peak amplitude was calculated by dividing the average amplitude of the 2^nd^ peak by that of the 1^st^.

Estimates of the readily releasable pool were determined by methods previously described^15,37,38^: In brief, EPSC amplitudes were measured by subtracting the baseline current just preceding an EPSC from the subsequent peak of the EPSC. EPSC amplitudes were then summed throughout the train stimulus to obtain a cumulative EPSC curve. For more detail on the general theory behind this method refer to published work^16^. In this study, a straight line was fitted to the final 7 points of the cumulative EPSC and back-extrapolated to the y-axis. This particular position (as marked by a dashed horizontal line in Fig. 7C) was chosen because the total probability for a vesicle to be released at that stimulus number (26^th^) amounts to 0.8 (80% of the releasable vesicles are released by the 26^th^ stimulus of the train^14^).

### Neuronal reconstructions and Sholl analysis

Neuronal morphology was reconstructed from confocal image stacks after filling recorded neurons with biocytin (#B4261, Sigma Aldritch). Dendritic length was calculated from reconstructed neuronal morphology using the Sholl Analysis function of the NeuronStudio software^39,40^. Branching points were calculated by analyzing the skeletonized neuronal morphologies using the Neuromantic software^41^.

### Urine odor exposures to induce c-Fos expression

Female mice underwent surgery for AOB Ct-b injections as described above. Unilateral tracer injections were performed at about P10 and P40. To prevent damage to the vomeronasal nerves and the AOB, injections were made rostral to the AOB, at a 45-degree angle. Two weeks after recovery, female mice were exposed for 30 minutes to urine pooled from group housed (2–3 per cage) sexually-mature males and females (3–4 months old). About 1 hour after odor exposure, mice were perfused and their brains processed for c-Fos immunohistochemistry in the PmCo. Mature females subjected to odor familiarization were exposed twice daily (10:00 am, 5 pm for 7 days) to an assigned individual male odor or home-cage bedding material prior to the final odor exposure.

To trigger c-Fos expression, the following stimuli included: (1) novel stimuli consisting of freshly collected individual male urine (nMU), (2) familiar stimuli derived from the same individual male used during odor familiarization (fMU), (3) unfamiliar stimuli (uMU) obtained from a different male and presented to previously familiarized females. Prior to the final odor exposures, female mice were removed from their home-cages, placed in clean bedding material for 2 hours and exposed to one of the above mentioned odor sources for 30 minutes. Different “stimulus male mice” were interchangeably used as sources for experienced or unexperienced urine odors to avoid bias due to individual differences. One hour after the end of this stimulation, female subjects were euthanized and brain tissue was dissected as described above. Mice exhibiting less than 100 Ct-b labeled cells in the PmCo (N = 2) were excluded from the analysis.

### Data analysis

Statistical tests included t-tests in the case of simple comparisons (indicated in the manuscript text) and F-tests (1 way or 2 way ANOVA) in all other analyses (see table including all statistical tests). Statistical significance was reached for an alpha error of 0.05 (p < 0.05) except in one case in which the same counts were used for two different comparisons (the “ctrl” and “MU” groups have been used both for the comparison shown in Fig. 8C but also for the one displayed in Fig. 8D). In this case the alpha error (α = 0.05) was set to 0.025 to adjust for multiple comparisons (adjusted alpha (α) = α/k, where k is the number of hypothesis tested: 0.05/2 = 0.025)^42^. The Tukey post-hoc test was used for multiple comparisons, and the alpha value was corrected accordingly in this last example. Categorical principal component analysis (CATPCA) was run using two factors and *varimax* rotation^43^ (SPSS software). For an exhaustive list of the statistical tests used, together with their results, see Table 2 (Supplementary information).

## Supplementary information

Supplementary information.
