# Supplementary material for: Gradual wiring of olfactory input to amygdala feedback circuits
Source: Sci Rep. 2020 Apr 3;10:5871. doi: 10.1038/s41598-020-62457-2 (PMC7125095; doi:10.1038/s41598-020-62457-2)
Supplement: Supplementary file 1 — Supplementary information. [file 41598_2020_62457_MOESM1_ESM.pdf]

Title: Gradual wiring of olfactory input to amygdala feedback circuits

Authors: Livio Oboti[1,2], Kate Sokolowski[1]

[1]Center for Neuroscience Research, Children's National Health System, Washington, DC, 20010, USA.

[2]Current address: Department of Behavioral Physiology, Humboldt Universität zu Berlin, Berlin, 10115, Germany.

Supplementary Information

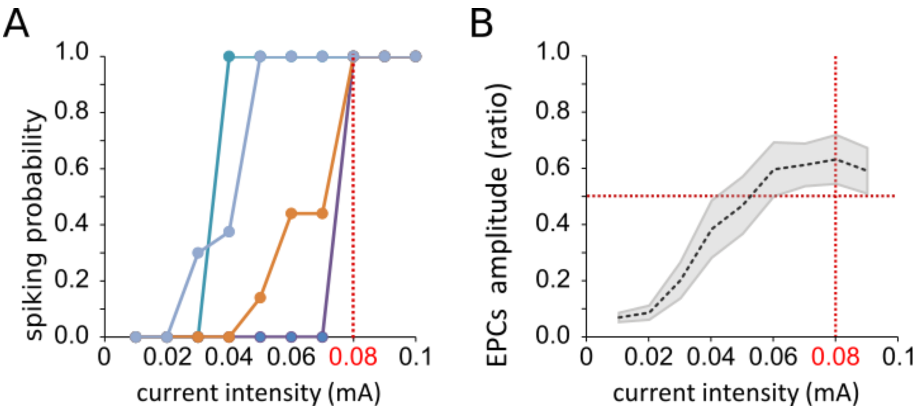

Figure S1: Current threshold for mature ACP excitation.

A) Spiking probability of ACP measured applying current stimulations of different intensity to PmCo layer I (cell attached mode). Response curves related to different cells are represented in different colors. B) Amplitude of evoked responses measured during intracellular recordings and electrical stimulation of PmCo layer I (shaded area represents SEM).

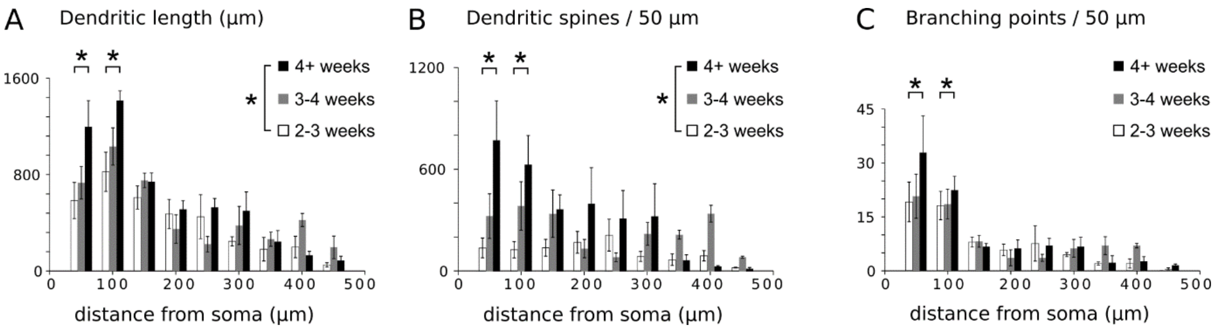

Figure S2: Analysis of ACP morphological parameters on different cellular domains. A) Age-dependent increase in dendritic length with no apparent bias for the proximal or distal dendritic domains. B) Changes of spine density across different dendritic domains at different stages. C) Age dependent changes in dendritic branching. Data are expressed as mean ± SEM and are referred to all neuronal processes sampled within a given radial distance from the soma. See Table 2 for the results of the statistical tests.

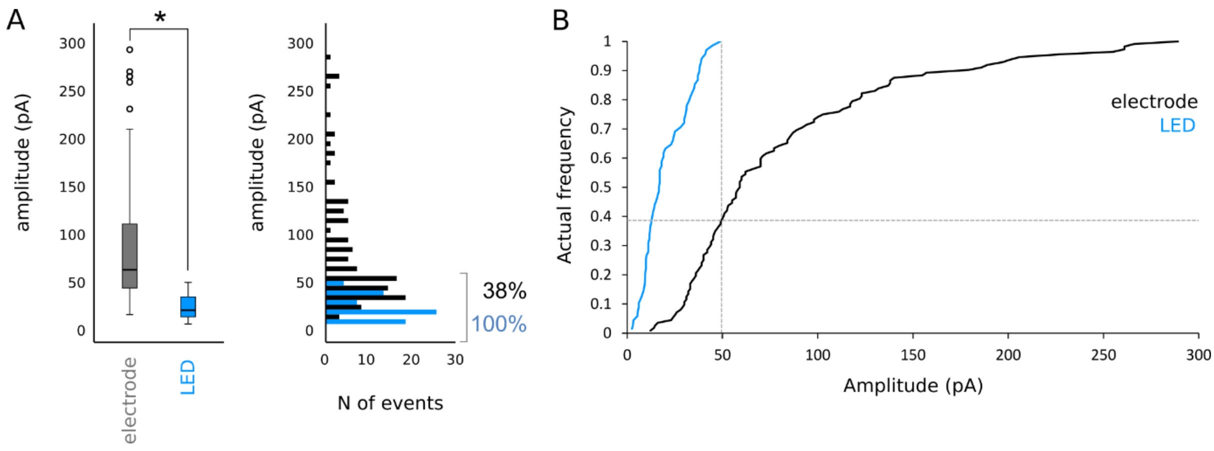

Figure S3: Amplitude of evoked electrode- and LED-induced responses. A) Box plot showing the amplitude comparison of currents evoked by the two types of stimuli. Currents evoked by electrode stimuli are higher in amplitude compared to LED-evoked stimuli (Wilcoxon, $p < 0.001$). Histogram shows the extent of overlap between the two amplitude ranges (all LED evoked currents are as large as

approximately 38% of those evoked by electrode stimulations). B) Cumulative amplitude distributions showing the overlapping amplitude ranges of the two response types: the maximum amplitude value of LED-evoked responses is close to 40% of the maximum response evoked by electrode stimulations. See Table 2 for the results of the statistical tests.

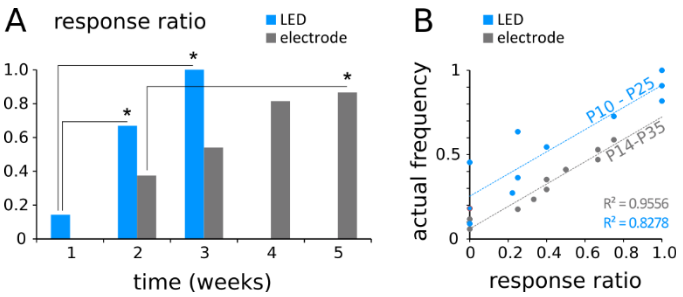

Figure S4: Weekly changes in ACP response ratios to electrode or LED evoked stimuli. A) Comparison of the gradual changes in the ratio of responsive vs non-responsive cells calculated after grouping by age (Figure 2F) in weeks. B) Cumulative distributions of response ratios to electrode and LED evoked stimuli show similar increase rates over time (age ranges for cumulative firing probability: electrode P14-P35, LED P10-25). See Table 2 for the results of the statistical tests.

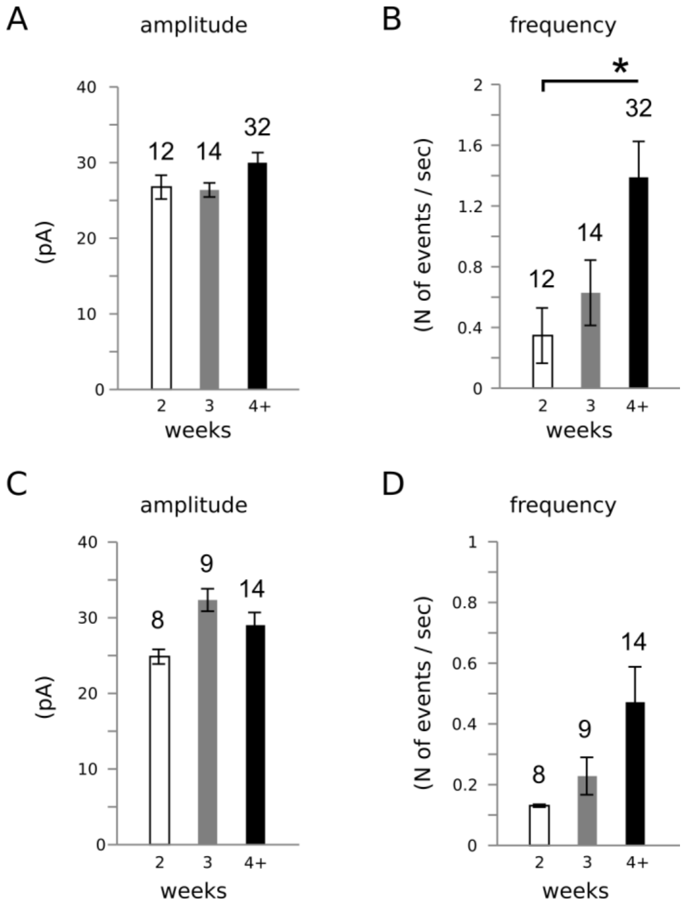

Figure S5: Changes in network spontaneous events across postnatal stages (larger samples). (A-B) Amplitude and frequency comparisons of EPSCs recorded in ACPs at 2-4 weeks and later stages. (C-D) Amplitude and frequency comparisons of IPSCs recorded in ACPs at 2-4 weeks and later stages. Data are expressed as mean ± SEM. See Table 2 for the results of the statistical tests.
